# Translational Biomedical Informatics in the Cloud: Present and Future

**DOI:** 10.1155/2013/658925

**Published:** 2013-03-17

**Authors:** Jiajia Chen, Fuliang Qian, Wenying Yan, Bairong Shen

**Affiliations:** ^1^Center for Systems Biology, Soochow University, Suzhou 215006, China; ^2^School of Chemistry and Biological Engineering, Suzhou University of Science and Technology, Suzhou 215011, China

## Abstract

Next generation sequencing and other high-throughput experimental techniques of recent decades have driven the exponential growth in publicly available molecular and clinical data. This information explosion has prepared the ground for the development of translational bioinformatics. The scale and dimensionality of data, however, pose obvious challenges in data mining, storage, and integration. In this paper we demonstrated the utility and promise of cloud computing for tackling the big data problems. We also outline our vision that cloud computing could be an enabling tool to facilitate translational bioinformatics research.

## 1. Introduction

The rate of accumulation of biomolecular data is increasing astonishingly. This information explosion is being driven by the development of low-cost, high-throughput experimental technologies in genomics, proteomics, and molecular imaging, amongst others. Success in the life sciences will depend on our ability to rationally interpret these large-scale, high-dimensional data sets into clinically understandable and useful information, which in turn requires us to adopt advances in informatics. Translational informatics, given the available data resources, is now evolving as a promising methodology that can drive the translation of laboratory data at the bench to health gains at the bedside. This “translation” involves correlating genotype with phenotype, which often requires dealing with information at all structural levels ranging from molecules and cells to tissues and organs, individuals to populations.

## 2. Translational Bioinformatics: Imperative to Collaborate

According to the scale of investigation, the fields of translational informatics can be roughly classified into four subdisciplines [1]: (1) bioinformatics (molecules and cells); (2) imaging informatics (tissues and organs); (3) clinical informatics (individuals); and (4) public health informatics (populations). Each of the subfields is directed at a particular level of research scale.  Table 1 outlines the spectrum of translational bioinformatics activities. The four subfields of translational bioinformatics are compared along several dimensions, including (1) areas of research purpose; (2) data types; (3) informatics tools to support practice.

Bioinformatics traditionally concerns applying computational approaches to the analysis of massive data from genomics, proteomics, metabolomics, and the other “-omic” subfields. Such research might help better comprehend the intricate biological details at molecular and cellular levels. Imaging informatics is focused on what happens at the level of tissues and organs. The essential informatics techniques to extract and manage the biological knowledge from images are summarized in Table 1. At the individual level, clinical bioinformatics is oriented to provide the technical infrastructure to understand clinical risk factors and pathophysiological mechanisms. As for public health informatics, the stratified population of patients is at the center of interest. Such research relies on informatics solutions to study shared risk factors for disease on a population level. 

At each of these levels, large amounts of experimental data are being generated. To fully understand a disease phenomenon, however, it is important to gather data at various levels and analyze them in an integrated fashion. While the four areas of research differ in their scientific foundations, they nevertheless share a core set of informatics methodologies, such as data acquisition systems, controlled vocabularies, knowledge representation, simulation and modeling, information retrieval, and signal and image processing, which provide a basis for their intersection.

## 3. Crisis Looms for Multidisciplinary Collaboration

The current push for personalized disease treatment is encouraging bioinformatics to seamlessly integrate data acquired from multiple levels of investigation, from molecular scale to organisms and tissues and further to individuals and populations. To achieve this goal, multidisciplinary collaboration between the fundamental aspects of translational informatics (e.g., bioinformatics, imaging informatics, clinical informatics, and public health informatics) has become essential. 

However, the large scale and high dimensionality of data have posed obvious challenges in data mining, storage, and integration. Traditionally, basic research, clinical research, and public health are seen as different worlds based on distinct or incompatible principles. Data transfer, access control, and model building rank are among the most pressing challenges.

## 4. Cloud Computing to the Rescue

Recent studies and commentaries [2–6] have proposed cloud computing as a solution that addresses many of the limitations mentioned above. Cloud computing is a relatively recent invention. It refers to a flexible and scalable internet infrastructure where processing and storage capacity are dynamically provisioned. The basic idea of cloud computing is to divide a large task into subtasks, which can then be executed on a number of parallel processors. A key technology with the cloud is the virtual machine (VM) that can be prepackaged with all software needed for a particular analysis.

Large utility-computing services have been emerging in the commercial sector, for example, the Amazon Elastic Compute Cloud (EC2) (http://aws.amazon.com/ec2/) [7], and noncommercial public cloud computing platforms also exist to support research, such as the IBM/Google Cloud Computing University Initiative [8] launched by Google and IBM.

Cloud computing infrastructures offer a new way of working. It features a special parallel programming model (e.g., MapReduce [9] designed by Google) to efficiently scale computation to many thousands of commodity machines. These commodity machines form a cluster that can be rented over the internet. Applications in the cloud have also benefited from hadoop (http://hadoop.apache.org/) [10], an open-source implementation of MapReduce. Since it is easy to fine tune and highly portable, Hadoop, together with MapReduce, has been widely used for large-scale distributed data analysis in both academy and industry.

Cloud computing infrastructures offer a highly flexible and economical means of working. Cloud computing provides scalable, flexible access to larger computer processing power and storage and avoids the fixed cost of capital investments in local computing infrastructures, computing maintenance, and personnel. The end users are essentially renting capacity on their demand [11].

Cloud computing allows the sharing of data in real-time collaboration with other users. It addresses one of the challenges related to transferring and sharing data. Researchers can store their data in the cloud with high availability. For example, Amazon web services provide free access to many useful data sets, for example, the Ensembl [12] and 1000 Genomes data [13]. In addition, the users can have thousands of on-demand powerful computers ready to run their analysis. To this end, cloud computing has the potential to facilitate large-scale efforts in translational data integration and analysis.

## 5. Translational Bioinformatics Research in the Cloud

There is considerable enthusiasm in the bioinformatics community to deploy open-source applications in the cloud. Various services provided by cloud-computing vendors are described below.

### 5.1. Cloud-Based Application in Bioinformatics

Numerous of studies have reported the successful application cloud computing in bioinformatics research. Most of these cloud computing applications deal with high-throughput sequence data analysis. CloudBLAST [14] is the first cloud-based implementation to solve sequence analysis problems. Other projects have since been launched on the cloud. Some initiatives have utilized preconfigured software on cloud systems to support large-scale sequence processing. Some tools are available for sequence alignment, short read mapping, SNP identification, genome annotation, and RNA differential expression analysis, amongst others (Table 2). Efforts in comparative genomics [15–20] and proteomics [21] have also incorporated the cloud to expedite their data processing.

### 5.2. Cloud-Based Application in Imaging Informatics

The volumes of high-resolution and dynamic imaging data can be estimated to reach petabytes, which indicates that the image reconstruction and analysis is computationally demanding. Cloud-computing is an obvious potential contributor to this end. Image clouds would enable multinational sharing of imaging data, as well as advanced analysis of imaging data away from its place of origin. 

Many studies have shown the utility of MapReduce for solving large-scale medical imaging problems in a cloud computing environment. For example, Meng et al. [22] developed an ultrafast and scalable image reconstruction technique for 4D cone-beam CT using MapReduce in a cloud computing environment. Avila-Garcia et al. [23] proposed a cloud computing-based framework for colorectal cancer imaging analysis and research for clinical use. Silva et al. [24] implemented a set of DICOM routers interconnected through a public cloud infrastructure to support medical image exchange among institutions.

Imaging clouds is also making unprecedentedly large-scale imaging research feasible. For example, Euro-Bioimaging [25], a pan-European research infrastructure project aims to deploy a distributed biological and biomedical imaging infrastructure in Europe in a coordinated and harmonized manner. It is expected to offer platforms for storing, remotely accessing, and post-processing imaging data on a large scale.

### 5.3. Cloud-Based Application in Clinical Informatics

A major challenge for clinical bioinformatics pertains to the accommodation of the range of heterogeneous data into a single, queryable database for clinical or research purposes. Electronic health record (EHR), an integrated clinical information storage systems, has recently emerged and stimulated increased research interest. EHR is a record in digital format that is capable of organizing clinical data by phenotypic categories. An ideal EHR provides complete personal health and medical summary by integrating personal medical information from different sources. The inclusion of genetic imaging and population-based information in EHR has the potential to provide patients with valuable risk assessment based on their genetic profile and family history and to carve a niche for personalized cancer management. 

The potential benefits of cloud computing facilitating EHR sharing and EHR integration have been realized. With cloud computing, EHR service could store data into cloud servers. In this way the resources could be flexibly utilized and the operation cost can be reduced. It is envisioned that through the internet or portable media, cloud computing can reduce electronic health record startup expenses, such as hardware, software, networking, personnel, and licensing fees and therefore will promise an explosion in the storage of personal health information online [26–29].

Many previous studies proposed different cloud-based frameworks in an attempt to improve EHR. Among them, Chen et al. [30] proposed a new patient health record access control scheme under cloud computing environments which allows accurate access to patient health record with security and is suitable for enormous multiusers. Chen et al. [31] proposed an EHR sharing and integration system in healthcare clouds. Doukas et al. [32] presented the implementation of a mobile system that enables electronic healthcare data storage, update and retrieval using cloud computing. Rolim et al. [33] proposed a cloud-based solution to automate processes for patients' vital data collection via a network of sensors connected to legacy medical devices and to deliver the data to a medical center's cloud for storage, processing, and distribution. The system provides users with real-time data accessibility labor work to collect, input, and analyze the information. Rao et al. [34] introduced a pervasive cloud-based healthcare application called Dhatri, which leveraged the power of cloud computing and mobile communications technologies to enable physicians to access real-time patient health information from remote areas. 

Besides academic researches described above, multiple commercial vendors are competing on this relatively new market. Many world-class commercial companies have heavily invested in the cloud, offering personal medical records services, such as Microsoft's HealthVault [35], which is currently the largest commercial personal health report platform.

### 5.4. Cloud-Based Application in Public Health Informatics

Public health informatics heavily relies on the data exchange between public health departments and clinical providers. However, public health's information technology systems lack the capabilities to accept the types of data proposed for exchange. Data silos across organizations and programs will present a set of challenges. With cloud services, however, public health applications, software systems, and services would be made available to health departments, therefore facilitating the exchange of specified types of data between different organizations. In addition, through remote hosting and shared computing resources, public health departments could overcome the problem of funding constraints and insufficient infrastructure for public health systems. 

## 6. Concerns and Challenges for the Biomedical Cloud

Cloud computing offers new possibilities for biomedical research, as data can now be easily accessed and shared. Despite the potential gains achieved, there are also several important issues to be addressed before the cloud computing can become more popular. The most significant concerns pertain to information security and data transfer bottlenecks.

### 6.1. Information Security and Privacy

Lately, many healthcare organizations are looking to move data and applications to a cloud environment. While this offers flexibility and easy access to computational resources, it also introduces security and privacy concerns, which are particularly evident in fields such as clinical informatics and public health informatics. Highly specialized data, such as clinical data from human studies, have exceptional security needs. Hosting such data on publicly accessible servers may increase the risk of security breaches. There are additional privacy concerns relating to personal information. Therefore these data must be posted according to privacy and security rules, such as the Health Insurance Portability and Accountability Act (HIPAA) [36]. For a biomedical cloud to be viable, a secure protection scheme will be necessary to protect the sensitive information of the medical record. For example, sensitive data will have to be encrypted before entering the cloud. Also, only authorized users are allowed to place and acquire sensitive security metadata in the cloud. More advanced encryption measures as well as access control schemes need to be deployed under cloud computing environments. 

So far, some research efforts have been made to build security and privacy architectures for biomedical cloud computing [37, 38]. Main cloud service providers (e.g., Amazon, Microsoft, and Google) have also made commitments to develop best practices to protect data security and privacy.

### 6.2. Data Transfer Bottlenecks

Another major obstacle to moving to the cloud is the time and cost of data transfer. Biomedical research institutions may need to frequently export or import large volumes of data (on the order of terabytes and soon to be petabytes) to and from the cloud. Given the size of the data set, one may find that there is a data transfer bottleneck. Networking bandwidth limitation causes delays in data transfer and incurs high bandwidth costs from service providers. Bandwidth costs might be low for smaller internet-based applications that are not data intensive. However, as applications continue to become more data intensive, these costs can quickly add up, making data transfer costs an important issue. For applications that require substantial data movement on a regular basis, cloud computing currently does not make economic sense.

## 7. Future Developments and Applications

As discussed above, the future of translational medical bioinformatics will depend on integration of diverse data types of patient characteristics. It is therefore crucial to develop an open, data-sharing environment. We suggest that future initiatives should include (1) development of standards to facilitate informational exchange, (2) integration of databases to allow cross-referencing of multilevel data.

### 7.1. The Need for Standardized Data Formats

Data exchange across the subfields of translational bioinformatics is often difficult because the data come from heterogeneous informatics platforms and are stored in different formats (e.g., numerical values, free text, and graphical and imaging material). The high dimensionality of potential data types mandates standards to represent data in a uniform manner. To work toward this goal, integrated medical/biological terminologies and ontologies have to be adopted, together with advanced semantic-based models and natural language processing (NLP) techniques to objectively describe medical and biomolecular findings. 

Numerous attempts have been made in developing standards for data integration in specialized domains. For example, minimum information about microarray experiment (MIAME) [39] is a standard developed to represent and exchange microarray data. In the field of imaging informatics, existing standards include the foundational model of anatomy clinical community, and digital imaging and communications in medicine (DICOM) [40]. Health level 7 (HL7), clinical data standards interchange consortium (CDISC), systematized nomenclature of medicine (SNOMED) and the international statistical classification of diseases and related health problems (ICD-10) represent the standard for clinical community.

These community-specific standards alone, however, are not sufficient to enable intercommunity data sharing. In this regard, the development of integrated standards will be essential. While it is unlikely to develop a single standard to cover all domains, semantic mapping between terminologies seems more practical. Several pioneering medical informatics projects are underway to define such intercommunity standard. For example, the ACGT project [41] launched by the European Union developed a set of methodological approaches as well as tools and services for semantic integration of distributed multilevel databases. 

### 7.2. The Need for Unified Databases

Currently different layers of biomedical data are stored within databases that are highly distributed, and often not interoperable. Even the databases that hold large data sets are often specialized and fragmented, obstructing the path to information sharing. We need database integration to allow cross-referencing of multilevel data for research or clinical purposes. Opportunities to develop integrated storage systems are increasing as a result of participatory initiatives. Funded by the US National Institutes of Health (NIH), many local platforms in the biomedical informatics space have been established to support data sharing, including informatics for integrating biology and the bedside (i2b2) [42], cancer biomedical informatics grid (caBIG) [43], and biomedical informatics research network (BIRN) [44]. 

NIH-funded i2b2 Center developed an open-source scalable informatics framework that integrates clinical research data from medical record and genomic data from basic science research. This platform helps better understand the genetic bases of complex diseases. To date, i2b2 has been deployed by over 70 sites internationally. caBIG aims to provide open source standards for data exchange and interoperability in cancer research. At the heart of the caBIG approach is a grid middleware infrastructure called caGrid. caGrid is a service-oriented platform that provides the tools for organizations to integrate data silos, securely share data, and compose analysis pipelines. caBIG enjoys widespread adoption throughout the cancer community. BIRN is an initiative funded by NIH to provide infrastructure, software tools, strategies, and advisory services for sharing biomedical research across disparate groups. These efforts contributed to the transfer and integration of distributed, heterogeneous and multilevel data across the major realms of translational bioinformatics.

## 8. Conclusion

Biomedical cloud, given the proper architecture, could integrate all the petabytes of available biomedical informatics data in one place and process them on a continuous basis. In this way, we would continuously observe the connections between genotypic profiles and phenotypic data. We can envision that the cloud-supported translational bioinformatics endeavors will promote faster breakthroughs in the diagnosis, prognosis, and treatment of human disease.

## Figures and Tables

**Table 1:** Spectrum of translational bioinformatics activities.

Subfields	Research purpose	Data types	Informatics tools
Bioinformatics	Sequencing Structure analysis Expression analysis Phylogenetic analysis Structure modeling	Sequence information Microarray Mass spectrum SNP Haplotypes	Pattern recognition [45–47] Data mining [48–50] Machine learning [47, 51, 52] Visualization [53–55] Automatic annotation [49, 55–58]

Imaging informatics	Image feature identification Image segmentation Image reconstruction Image annotation Image indexing Image visualization	DICOM JPEG TIFF PNG GIF BMP	Content-based image retrieval [59, 60] Natural language processing [61, 62]

Clinical informatics	Clinical decision support Clinical information access Electronic patient record system Disease reclassification	Clinical laboratory results Physical examination Symptoms and signs Patient history Prescriptions	Probabilistic decision-making [63] Expert reasoning system [64–66] Assessment and validation vocabularies [67, 68] Text-parsing tools [69]

Public health informatics	Tracking of infectious diseases Assessment clinical interventions Monitoring disease risk factors	PHCDM-based health information	Access control [70, 71] Information security technology [37, 72] Semantic and syntactic standards [73] Structured data-collection techniques [74, 75]

**Table 2:** Application of cloud computing in bioinformatics research.

Software	Website	Description	Reference
ArrayExpressHTS	http://www.ebi.ac.uk/Tools/rwiki/	RNA-Seq data processing and quality assessment	[76]
Bioscope	http://www.lifescopecloud.com/	Reference-based read mapping	[77]
Cloud-MAQ	http://sourceforge.net/projects/cloud-maq/	Read mapping and assembly	[78]
CloudAligner	http://sourceforge.net/projects/cloudaligner/	Read mapping	[79]
CloudBurst	http://cloudburst-bio.sourceforge.net/	Reference-based read mapping	[6]
CloudCoffee	http://www.tcoffee.org/homepage.html	Multiple sequence alignment	[20]
Contrail	http://contrail-bio.sourceforge.net/	De novo read assembly	[80]
Crossbow	http://bowtie-bio.sourceforge.net/crossbow/	Read mapping and SNP calling	[4]
FX	http://fx.gmi.ac.kr/	RNA-Seq data analysis for gene expression levels and genomic variant calling	[81]
GeneSifter	http://www.geospiza.com/Products/AnalysisEdition.shtml	Customer-oriented NGS data analysis services	[82]
Myrna	http://bowtie-bio.sf.net/myrna/	Differential expression analysis for RNA-Seq data	[83]
PeakRanger	http://www.modencode.org/software/ranger/	Peak caller for ChIP-Seq data	[84]
Roundup	http://rodeo.med.harvard.edu/tools/roundup/	Optimized computation for comparative genomics	[19]
SeqMapReduce	http://www.seqmapreduce.org/	Read mapping	[85]
YunBe	http://tinyurl.com/yunbedownload/	Gene set analysis for biomarker identification	[86]
